# Urinary Exosomal Long Noncoding RNA TERC as a Noninvasive Diagnostic and Prognostic Biomarker for Bladder Urothelial Carcinoma

**DOI:** 10.1155/2022/9038808

**Published:** 2022-01-25

**Authors:** Chen Chen, Anquan Shang, Zujun Sun, Yuting Gao, Jingjuan Huang, Yili Ping, Wenjing Chang, Chenzheng Gu, Junjun Sun, Ping Ji, Yi Yuan, Renquan Lu, Dong Li

**Affiliations:** ^1^Department of Laboratory Medicine, Shanghai Tongji Hospital, School of Medicine, Tongji University, Shanghai 200065, China; ^2^Department of Clinical Laboratory, Fudan University Shanghai Cancer Center, Shanghai, China; ^3^Department of Oncology, Shanghai Medical College, Fudan University, Shanghai, China

## Abstract

**Purpose:**

Bladder cancer is one of the most common urological malignancies worldwide, and approximately 90% of bladder cancer cases are histologically typed as bladder urothelial carcinoma (BLCA). Exosomes are 30 to 200 nm extracellular vesicles that transport microRNAs, long noncoding RNAs (lncRNAs), mRNAs, circular RNAs, and proteins across tissues and through circulation. Urinary exosomes may contain genetic information from tumor cells. Herein, we explored the clinical significance of urinary exosomal lncRNA telomerase RNA component (*TERC*) levels to provide an urgently needed diagnostic and prognostic biomarker for BLCA.

**Materials and Methods:**

In this study, we used RNA-sequencing of samples from four BLCA patients and three healthy controls to identify that *TERC* was differentially expressed in urinary exosomes. We then used quantitative PCR in different types of clinical samples to validate the biomarker and analyzed results using receiver operating characteristic curves.

**Results:**

We found that *TERC* was significantly upregulated in urinary exosomes from BLCA patients compared with those from healthy controls (*P* < 0.0001). Urinary exosomal *TERC* showed higher sensitivity (78.65%) and accuracy (77.78%) than existing indicators including nuclear matrix protein-22 and urine cytometry. Using the cut-off value 4.302, the area under the curve for urinary exosomal *TERC* was 0.836 (95% confidence interval: 0.768–0.891, *P* < 0.0001). Furthermore, this noninvasive assay could distinguish low-grade and high-grade tumors (*P* = 0.0153).

**Conclusions:**

*TERC* is enriched in urinary exosomes from BLCA patients. Urinary exosomal *TERC* could become a diagnostic and prognostic biomarker for BLCA that allows clinicians to realize noninvasive detection of BLCA.

## 1. Introduction

Globally, bladder cancer is one of the most common urological malignancies, with approximately 90% of cases histologically defined as bladder urothelial carcinoma (BLCA). In 2020, the incidence of bladder cancer ranked tenth among all malignant tumors, and its incidence and mortality ranked second among male urinary system malignancies [1]. The gold standard for diagnosing bladder cancer remains tissue biopsy *via* cystoscopy. The colloidal gold immunochromatographic assay (GICA) for nuclear matrix protein- (NMP-) 22 and bladder tumor antigen are clinical methods approved by the US Food and Drug Administration; however, their sensitivity is too low, and hematuria always leads to false positive results [2–4]. Thus, there is an urgent need for a more meaningful diagnostic index for bladder cancer that balances sensitivity and specificity.

Exosomes are potential diagnostic samples from liquid biopsy because they can transport mRNAs, microRNAs, long noncoding RNAs (lncRNAs), circular RNAs, lipids, and proteins throughout bodily fluids [5, 6]. The exosomal lipid bilayer protects the contents from RNases and proteases [7]. lncRNAs are defined as transcripts of >200 nucleotides and have been demonstrated to play critical roles in many biological and pathological processes rather than being “transcriptional noise” [8]. It has been reported that exosomes can selectively package certain lncRNAs that are abundantly present in exosomes [9, 10]. BLCA tumor cells continuously release exosomes that contain lncRNAs into urine. Urinary exosomal lncRNAs can directly reflect the malignant state and have the possibility to be diagnostic and prognostic biomarkers for BLCA [11–14].

Using deep RNA-sequencing of urinary exosomes, we found that the lncRNA telomerase RNA component (*TERC*, 451 nt in length) was differently expressed between four BLCA patients and three healthy controls. *TERC* and telomerase reverse transcriptase (TERT) compose telomerase. In most human tumors, telomerase is activated to maintain telomere length, extend lifespan, and reduce apoptosis of tumor cells [15]. Many previous studies have reported the roles of *TERC* in telomere biology and in promoting tumorigenesis and inflammation, but there are still few reports regarding the diagnostic and/or prognostic value of exosomal *TERC* expression [16–19].

## 2. Materials and Methods

### 2.1. Patients and Volunteers

Samples were collected from BLCA patients with histopathologically confirmed diagnosis between October 2019 and May 2021 at Shanghai Tongji Hospital and Fudan University Shanghai Cancer Center. The clinical information of patients is provided in Supplementary File 1. In total, healthy participants were sought. The Ethics Committee of Shanghai Tongji Hospital (No. 2021-KYSB-064) and Fudan University Shanghai Cancer Center (No. 050432-4-1911D) authorized this study.

### 2.2. Isolation of Exosomes

Exosomes for sequencing were isolated from 150 mL of urine using differential centrifugation. The isolation procedure was as follows, taking the supernatant from each step: urine was centrifuged at 600 × *g* for 20 min, 2000 × *g* for 30min, and 10000 × *g* for 1h and then filtered through a 0.22 *μ*m filter. Finally, ultracentrifugation at 100000 × *g* for 2h was performed, and the supernatant was discarded. All centrifugation steps were performed at 4°C. Exosome pellets were resuspended in 50 *μ*L of 0.22 *μ*m filtered phosphate-buffered saline.

Exosomes used for validation were isolated from human urine in strict accordance with the instructions of the exosome extraction kit (BestBio, Shanghai, China). 8 mL of urine was centrifuged at 3000 × *g* for 15 min at 4°C, and the supernatant was then centrifuged at 10000 × *g* for 20 min at 4°C. The supernatant was then mixed with solution A and stored 4°C overnight. The mixture was centrifuged at 10000 × *g* for 1 h at 4°C and exosome pellets were resuspended in 500 *μ*L TRIzol-LS (Life Technologies, Carlsbad, CA, USA).

### 2.3. Transmission Electron Microscopy (TEM)

Exosome suspensions were placed on 400-mesh carbon-coated copper grids and negatively stained with 2% phosphotungstic acid solution. TEM was performed to view and capture images (Thermo Fisher Scientific, Waltham, MA, USA).

### 2.4. Nanoparticle Tracking Analysis (NTA)

The NanoSight LM10 system (Malvern Instruments Ltd., Malvern, UK) was used to detect the concentration and size distribution of particles.

### 2.5. Western Blot Analysis

Exosome suspensions were mixed with the appropriate volume of 5x SDS loading buffer, the mixture was boiled for 15 min at 100°C, and then, the supernatant was collected for western blot analysis. The primary antibodies included anti-TSG101 (Abcam, Cambridge, UK), anti-HSP70, anti-Annexin V, and anti-CD9 (all from Cell Signaling Technology, Danvers, MA, USA). The secondary antibody used was peroxidase-conjugated goat antirabbit IgG (Millipore, Burlington, MA, USA).

### 2.6. RNA-Sequencing

RNA-sequencing (RNA-seq) was performed by Shanghai Kangcheng Biotechnology Company (Shanghai, China) using an Illumina Novaseq 6000 system (San Diego, CA, USA). Differences in transcript levels were analyzed and used to screen out the differentially expressed lncRNAs between the BLCA patient and healthy control groups. The comparison scheme was fold change > 1.5, *P* value (*F* test) ≤ 0.05, and mean FPKM ≥ 0.5 in each sample.

### 2.7. RNA Extraction and Real-Time Quantitative PCR (qPCR)

TRIzol-LS reagent was used to extract total exosomal RNA, cDNA was synthesized using the PrimeScript™ RT reagent kit with gDNA Eraser (Takara, Dalian, Japan), and qPCR was performed using the TB Green Premix Ex Taq II (Tli RNaseH Plus) (Takara) on an Applied Biosystems 7300 real-time PCR system (Waltham, MA, USA).  Table 1 shows the sequences of primers used in this study.

### 2.8. GICA and Enzyme-Linked Immunosorbent Assay (ELISA) Analyses of NMP-22

Urine exosomal NMP-22 was detected using the Alere NMP22® BladderChek® Test based on GICA and ELISA kits from Hualian Biotechnology (Wuhan, China). The assays were performed in accordance with the kit instructions.

### 2.9. Statistical Analysis

Data are presented as mean ± SD. The Mann–Whitney *U* test, Pearson's *χ*^2^ test, and Pearson's correlation analysis were used to assess differences or correlation. *P* < 0.05 was considered statistically significant. Receiver operating characteristic (ROC) curves were used to determine the sensitivity and specificity of indicators. All statistical analyses were performed using GraphPad Prism 8 (GraphPad Software, Inc., Sand Diego, CA, USA) and MedCalc version 15.2.2 (MedCalc Software Ltd., Ostend, Belgium). Analysis of Kaplan–Meier survival curves was performed using data from the PrognoScan database for BLCA (GSE13507).

## 3. Results

### 3.1. Identification of Exosomes Derived from Urine Samples

**Figure 1:**
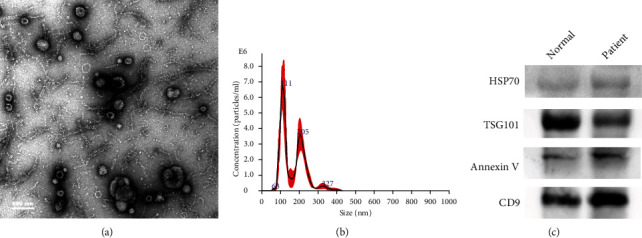
Characterization of urinary exosomes. (a) Transmission electron microscopy image of exosomes isolated from urine; scale bar: 100 nm. (b) Particles were observed in the size range of 50 to 200 nm by nanoparticle tracking analysis. (c) Western blot analysis of the exosomal protein markers HSP70, TSG101, Annexin V, and CD9. Exosomes were derived from healthy controls and bladder urothelial carcinoma patients.

According to the 2018 checklist for minimum information for studies of extracellular vesicles (MISEV2018) [20], we used a combination of TEM, NTA, and western blot analysis to identify exosomes. TEM showed that urinary exosomes were of round shape with cup-like concavity (Figure 1(a)). The size distributions of exosomes were analyzed by NTA. As shown in Figure 1(b), the size (diameter) of urinary exosomes ranged from approximately 50 to 200 nm (mean: 165.2 nm). Positive expression of four exosomal protein markers was confirmed by western blot analysis (Figure 1(c)) including of two cytosolic proteins (TSG101 and Annexin V) and two transmembrane proteins (HSP70 and CD9). Only strictly identified urine exosomes were used for further analysis and research [21].

### 3.2. RNA-Sequencing of Urinary Exosomes

**Figure 2:**
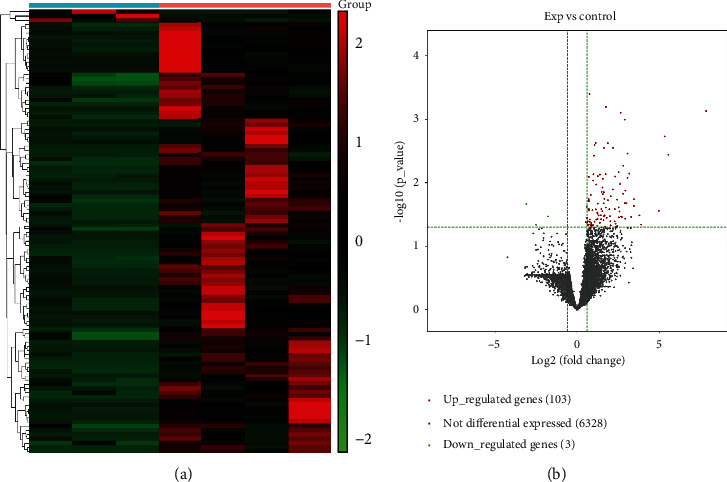
Heat map and volcano plot of differentially expressed long noncoding RNAs (DE-lncRNAs) from urinary exosomes. (a) Heat map of all DE-lncRNAs from urinary exosomes of healthy controls and bladder urothelial carcinoma (BLCA) patients. Results of the differential analysis are shown (*P* ≤ 0.05), with color from green to red indicating low to high fragments per kilobase of exon per million mapped fragments (FPKM) of DE-lncRNAs. (b) Volcano plot of RNA-sequencing results. Green dots represent downregulated genes and red dots represent upregulated genes.

To identify differentially expressed BLCA-specific molecules, we performed RNA-sequencing on urine exosomes from four BLCA patients and three healthy controls [22]. The results revealed 106 differentially expressed lncRNAs between urine exosomes from BLCA patients and healthy donors, among which 103 were upregulated and three were downregulated (Figures 2(a) and 2(b)). Among the upregulated lncRNAs, we found high abundance of *TERC*, which has been previously reported to be related to “tumor growth.”

### 3.3. Increased *TERC* Expression in Urine Exosomes from BLCA Patients

**Figure 3:**
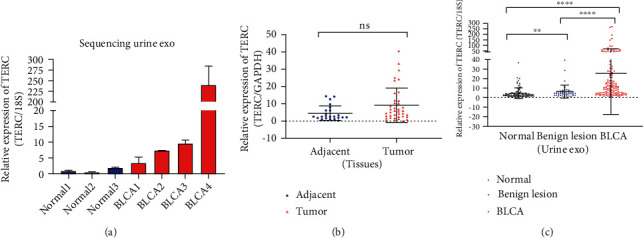
Relative telomerase RNA component (TERC) expression levels in bladder urothelial carcinoma (BLCA) patients. (a) TERC expression levels in urinary exosome samples from the three healthy controls and four BLCA patients used for RNA-sequencing are shown as mean ± SD. (b, c) Real-time quantitative PCR detection of TERC in different sample types including urine exosomes and tissues (ns: *P* > 0.05, ^∗∗^*P* < 0.01, ^∗∗∗∗^*P* < 0.0001). Relative expression levels in tissues were normalized to *GAPDH*, while *18S* was used as the internal reference gene in exosomes. Groups were compared with the nonparametric Mann–Whitney *U* test.

We performed qPCR to evaluate relative *TERC* expression in different types of clinical samples. We found that *18S* levels were more stable and abundant in urinary exosomes than the conventional housekeeping gene *GAPDH* and was more suitable as an internal reference gene for lncRNAs in exosomes. The absolute expression levels of genes are listed in Supplementary File 2. First, we detected *TERC* expression in urine exosomes from the three healthy controls and four BLCA patients used for sequencing (Figure 3(a)). In this small sample, we verified that *TERC* expression was higher in urinary exosomes from BLCA patients than from healthy controls, which was consistent with the RNA-sequencing results.

*TERC* was then detected in urine exosomes from 94 healthy controls, 46 patients with urinary benign lesions, and 128 BLCA patients (Figure 3(c)). The findings suggested that urinary exosomal *TERC* expression in BLCA patients was significantly higher compared with that in healthy controls and patients with urinary benign lesions (*P* < 0.0001), indicating that urinary exosomal *TERC* may have diagnostic value for BLCA. As shown in Figure 3(b), there was no difference between *TERC* expression of tumors (*n* = 39) and that of adjacent tissues (*n* = 23).

### 3.4. Urinary Exosomal *TERC* Had Better Diagnostic Value than NMP-22 (ELISA and GICA) and Urine Cytology

**Figure 4:**
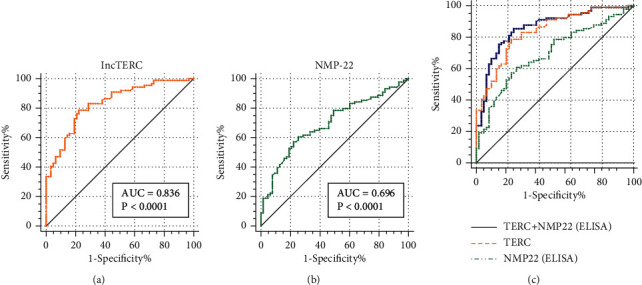
Diagnostic ability of exosomal telomerase RNA component (TERC) and nuclear matrix protein- (NMP-) 22 for bladder urothelial carcinoma (BLCA). (a–c) Receiver operating characteristic curve analysis of exosomal TERC and NMP-22 (ELISA) and the combination of these indicators revealed area under the curve values of 0.836, 0.696, and 0.861.

We examined *TERC* levels in urine exosomes from 89 BLCA patients and 63 healthy controls to determine its diagnostic utility. ROC curve analysis using the cut-off value 4.302 showed that *TERC* was a promising diagnostic biomarker with an area under the curve (AUC) of 0.836 (95% confidence interval [CI]: 0.768–0.891, *P* < 0.0001). The sensitivity and specificity of *TERC* were 78.65% and 77.78%, respectively (Figure 4(a)). Meanwhile, we detected NMP-22 from urine samples using ELISA; the results are listed in Supplementary File 2. The AUC, sensitivity, and specificity of NMP-22 (ELISA) were 0.696 (95% CI: 0.616–0.768, *P* < 0.0001), 60.67%, and 74.6%, respectively (Figure 4(b)). The AUC of combining the indicators reached 0.861 (95% CI: 0.795–0.911) (Figure 4(c)). Additionally, we analyzed NMP-22 (GICA) and urine cytology in 128 patients and 94 healthy controls. The results illustrated the high specificities of NMP-22 (GICA) and urine cytology (96.809% and 98.936%, respectively), but their sensitivities (31.25% and 43.75%, respectively) were much lower than that of urine exosomal *TERC* (78.65%) (Tables 2 and 3). In summary, these results demonstrated that urinary exosomal *TERC* had better diagnostic abilities than the existing indicators (NMP-22 [ELISA and GICA] and urine cytology).

### 3.5. Urine Exosomal *TERC* Was Associated with Tumor Grade, and Bioinformatics Analysis Showed Prognostic Predictive Value for BLCA

**Figure 5:**
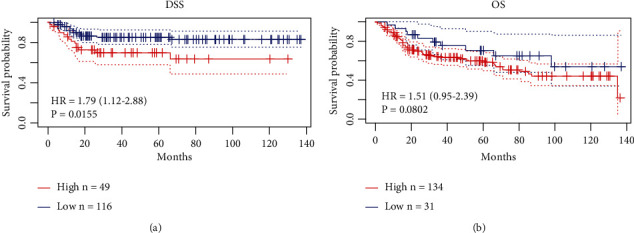
Analysis of Kaplan–Meier survival curves from the PrognoScan database. (a) The disease-specific survival (DSS) curve of TERC (hazard ratio [HR]: 1.79, 95% confidence interval [CI]: 1.12–2.88, *P* = 0.0155). (b) The overall survival (OS) curve of TERC (HR: 1.51, 95% CI: 0.95–2.39, *P* = 0.0802). HR > 1 indicates a risk factor associated with poor patient outcomes.

Patients' clinical characteristics including age, sex, TNM stage, histological grade, metastasis, and recurrence are summarized in Table 4. Pearson's *χ*^2^ test was performed to explore associations between exosomal *TERC* levels and clinical pathological characteristics. Patients were divided into the low- (*n* = 64) and high-expression (*n* = 64) groups, using the median value of *TERC* expression as the cut-off value. The findings indicated that the level of *TERC* expression was associated with tumor grade (*P* = 0.0153), while other clinical features such as metastasis and recurrence were not significantly correlated with *TERC*. Thus, we conclude that high TERC expression is associated with BLCA disease progression. Meanwhile, we analyzed Kaplan–Meier curves of disease-specific survival (DSS) and overall survival (OS) according to *TERC* expression using the PrognoScan database (http://dna00.bio.kyutech.ac.jp/PrognoScan/index.html) based on GSE13507. The hazard ratios (HRs) for DSS and OS were 1.79 and 1.51, respectively (Figures 5(a) and 5(b)). These results indicated that urinary exosomal *TERC* was a significant risk predictor with the potential to be a prognostic biomarker for BLCA.

^a^ *P* < 0.05, Pearson's *χ*^2^ test for the variables above in patients.

### 3.6. Urine Exosomal *TERC* Expression Was Not Correlated with Paired Tissues, and It Stably Exists after Freeze-Thaw Cycles due to Protection from the Lipid Bilayer

**Figure 6:**
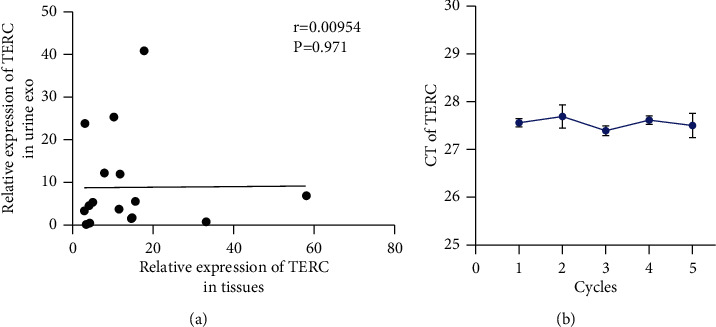
Correlation analysis of telomerase RNA component (TERC) levels in different sample types and its stability in exosomes. (a) TERC expression in tissues was uncorrelated with that in urine exosomes (*r* = 0.00954, *P* = 0.971). (c) Exosomal TERC levels after one to five freeze-thaw cycles of urine samples. Results are presented as mean ± SD.

To investigate the relationship in *TERC* expression between different types of samples, *TERC* levels were measured by qPCR in paired tissues and urine exosomes from BLCA patients [23]. Pearson's correlation analysis demonstrated that urine exosomal *TERC* levels were not correlated with the *TERC* levels in corresponding tissues; the correlation coefficients (*r*) were 0.00954 (Figure 6(a)).

Additionally, we divided urine samples into five aliquots of equal volume and placed each at −80°C for one to five freeze-thaw cycles [24]. The results revealed that exosomal *TERC* levels were stable after multiple freeze-thaw cycles (Figure 6(b)). This suggested that storing urine at −80°C even with multiple freeze-thaw cycles will not affect exosomal *TERC*, which was superior to detecting *TERC* in urine sediment cells [25, 26]. In summary, urinary exosomal *TERC* has the potential to be a stable and noninvasive diagnostic and prognostic biomarker for BLCA.

## 4. Discussion

Bladder cancer is one of the most frequent malignancies of the urinary system, with 90% of cases pathologically classified as BLCA. The cause of BLCA tumorigenesis is currently unclear and may include smoking, repeated infection, exposure to compounds, or genetic factors [27, 28]. The gold standard for diagnosing bladder cancer is pathological tissue biopsy, which is invasive and uncomfortable. This study introduced a novel assay that does not require expedited or other special handling procedures, and an 8 mL urine sample is more than enough for the analysis.

We identified that the lncRNA *TERC* was differentially upregulated in urinary exosomes of BLCA patients by RNA-sequencing. The important components of the telomerase are TERT and *TERC*. As previously reported, identifying *TERT* promoter mutations in the urine may substantially aid in the early detection of bladder cancer [29, 30]. However, exosomal *TERC* has thus far not been investigated as a diagnostic or prognostic biomarker for bladder cancer.

We found that *TERC* expression levels were not significantly different between tumor and adjacent nontumor tissues from BLCA patients but were significantly upregulated in urinary exosomes from BLCA patients compared with those from healthy controls. Furthermore, urinary exosomal *TERC* expression could distinguish between urinary benign diseases (inflammation, stones, and obstruction) and bladder cancer (*P* < 0.0001). *TERC* can circulate in whole body fluids under the envelopment of exosomes. Additionally, *TERC* could significantly distinguish high- versus low-grade BLCA (*P* = 0.0153), and Kaplan–Meier survival curves demonstrated its prognostic value. Importantly, multiple freeze-thaw cycles of urine did not affect the stability of exosomal *TERC*.

The exosome is the star molecule of liquid biopsy, and research into them is still in the early stages. Our findings are limited by current science and technology in terms of achieving rapid and efficient detection of exosomal RNA. To extract urinary exosomes, we employ the polyethylene glycol (PEG) precipitation technique, which normally involves around 10 hours of pretreatment (centrifugation and resting) and 1 hour of centrifugation at 10000 × *g*, and the exosome precipitation will be achieved using a standard high-speed centrifuge. Although costly ultracentrifuges are not needed, they still have the drawback of being time-consuming and multistep. At this stage, urine exosomal *TERC* can only be used as an adjunct to cystoscopy, with diagnostic efficacy superior to existing NMP-22, BTA, and other indicators, allowing for patient review and follow-up. From experimental research to clinical application, not only medical but also engineering, science, and other interdisciplinary collaborations are required, and completely automated batch detection of urine exosomal *TERC* is still a long way off.

In the future, more data on the detection of urine exosomal *TERC* will be collected via multicenter and large sample studies, and patients will be followed for a longer period of time to validate its diagnostic and prognostic value.

## 5. Conclusions

In summary, the long noncoding RNA *TERC* was first found in urine exosomes in this article, and we demonstrated that urinary exosomal *TERC* can be used as a diagnostic and prognostic biomarker for BLCA with the added benefit of reducing unnecessary injuries from tissue biopsies. This study provides a convenient, stable, repeatable, and noninvasive index for clinicians and BLCA patients. Thus, our discovery will have profound implications for diagnosis and monitoring of bladder urothelial carcinoma.

## Figures and Tables

**Table 1:** Primer sequences are listed in the table.

TERC	Forward	5′-GTGGTGGCCATTTTTTGTCTAAC-3′
	Reverse	5′-TGCTCTAGAATGAACGGTGGAA-3′
GAPDH	Forward	5′-TGATGACATCAAGAAGGTGG-3′
	Reverse	5′-TTGTCATACCAGGAAATGAGC-3′
18S	Forward	5′-CGTTCTTAGTTGGTGGAGCG-3′
	Reverse	5′-CGCTGAGCCAGTCAGTGTAG-3′

**Table 2:** Statistics of NMP-22 (GICA) and urine cytology in BLCA patients and normal individuals.

		BLCA	Normal
NMP-22 (GICA)	Positive	40	3
	Negative	88	91
Urine cytology	Positive	56	1
	Negative	72	93

**Table 3:** Diagnostic efficacy of NMP-22 (GICA) and urine cytology.

	NMP-22 (GICA)	Urine cytology
Sensitivity	31.250%	43.750%
Specificity	96.809%	98.936%
Positive likelihood ratio	9.792	41.125
Negative likelihood ratio	0.710	0.569
Positive predictive value	93.023%	98.246%
Negative predictive value	50.838%	56.364%

**Table 4:** The characteristics of bladder urothelial carcinoma patients.

Variables	Total	Expression of TERC		*χ* ^2^	*P* value
		High	Low		
Age				0.82	0.3651
≤60	49	22	27		
>60	79	42	37		
Sex				3.099	0.0783
Male	98	44	54		
Female	30	19	11		
TNM				1.315	0.2515
Ta~T1	105	50	55		
T2~T4	23	14	9		
Grade				5.88	0.0153^a^
Low	20	5	15		
High	108	59	49		
Lymphatic metastasis				1.026	0.3110
Negative	110	53	57		
Positive	18	11	7		
Recurrence				1.233	0.2668
Primary	103	54	49		
Recurrent	25	10	15		

## Data Availability

The datasets during and/or analyzed during the current study are available from the corresponding authors on reasonable request.

## Supplementary Materials

Supplementary 1 Supplementary File 1: the clinical information of patients.

Supplementary 2 Supplementary File 2: the absolute levels of genes and NMP-22 (ELISA).
