# Supplementary material for: Urinary Exosomal Long Noncoding RNA TERC as a Noninvasive Diagnostic and Prognostic Biomarker for Bladder Urothelial Carcinoma
Source: J Immunol Res. 2022 Jan 25;2022:9038808. doi: 10.1155/2022/9038808 (PMC8811540; doi:10.1155/2022/9038808)
Supplement: Supplementary 1 — Supplementary File 1: the clinical information of patients. [file 9038808.f1.docx]

| number | age | sex | recurrence | TNM | Grade | Lymphatic metastasis | NMP22 | urine cytology |
| --- | --- | --- | --- | --- | --- | --- | --- | --- |
| 1 | 42 | M | Primary | Ta | low | （-） | （＋） | （-） |
| 2 | 53 | M | Primary | T1 | high | （-） | （＋） | （-） |
| 3 | 62 | M | Primary | Ta | low | （-） | （-） | （-） |
| 4 | 84 | M | Recurrent | T1 | high | （-） | （＋） | （＋） |
| 5 | 68 | M | Primary | T1 | high | （-） | （-） | （-） |
| 6 | 72 | M | Recurrent | T1 | high | （-） | （＋） | （＋） |
| 7 | 68 | M | Primary | T1 | high | （-） | （-） | （-） |
| 8 | 67 | M | Primary | T1 | high | （-） | （＋） | （-） |
| 9 | 54 | M | Primary | T1 | high | （-） | （-） | （-） |
| 10 | 48 | M | Primary | T1 | high | （-） | （-） | （-） |
| 11 | 50 | M | Primary | T1 | high | （-） | （-） | （＋） |
| 12 | 56 | M | Primary | T1 | high | （-） | （-） | （-） |
| 13 | 49 | M | Primary | T1 | low | （-） | （-） | （-） |
| 14 | 67 | M | Primary | Ta | low | （-） | （-） | （-） |
| 15 | 44 | M | Primary | T2 | high | （-） | （＋） | （＋） |
| 16 | 71 | M | Recurrent | T1 | high | （-） | （＋） | （＋） |
| 17 | 74 | M | Primary | T1 | high | （＋） | （-） | （＋） |
| 18 | 72 | M | Recurrent | T2 | high | （-） | （-） | （-） |
| 19 | 69 | M | Primary | T1 | high | （-） | （＋） | （＋） |
| 20 | 68 | M | Primary | T1 | high | （-） | （-） | （＋） |
| 21 | 50 | M | Primary | T2 | high | （＋） | （＋） | （＋） |
| 22 | 76 | M | Recurrent | T1 | low | （-） | （-） | （-） |
| 23 | 64 | M | Recurrent | T1 | high | （＋） | （＋） | （＋） |
| 24 | 70 | M | Recurrent | T1 | high | （-） | （-） | （＋） |
| 25 | 73 | M | Primary | Ta | high | （-） | （＋） | （＋） |
| 26 | 70 | M | Primary | T1 | high | （-） | （＋） | （＋） |
| 27 | 49 | M | Primary | T1 | high | （-） | （-） | （-） |
| 28 | 59 | M | Primary | T2 | high | （+） | （-） | （-） |
| 29 | 53 | M | Primary | T1 | high | （-） | （-） | （＋） |
| 30 | 72 | M | Primary | T1 | high | （-） | （-） | （-） |
| 31 | 75 | M | Primary | T1 | high | （-） | （-） | （-） |
| 32 | 49 | M | Primary | T1 | high | （-） | （-） | （-） |
| 33 | 54 | M | Recurrent | T1 | low | （-） | （-） | （-） |
| 34 | 61 | M | Primary | T1 | high | （-） | （-） | （＋） |
| 35 | 45 | M | Recurrent | T1 | high | （-） | （＋） | （-） |
| 36 | 55 | M | Primary | T1 | high | （-） | （＋） | （-） |
| 37 | 65 | M | Primary | T1 | high | （-） | （-） | （＋） |
| 38 | 58 | M | Primary | T3 | high | （-） | （-） | （-） |
| 39 | 58 | M | Recurrent | T1 | low | （-） | （＋） | （-） |
| 40 | 64 | M | Primary | T1 | high | （-） | （＋） | （-） |
| 41 | 57 | M | Primary | T1 | high | （-） | （＋） | （＋） |
| 42 | 60 | M | Primary | T1 | low | （-） | （-） | （-） |
| 43 | 56 | M | Primary | T2 | high | （-） | （-） | （-） |
| 44 | 63 | M | Primary | T1 | high | （-） | （-） | （-） |
| 45 | 63 | M | Primary | T1 | high | （-） | （-） | （＋） |
| 46 | 71 | M | Primary | T1 | high | （-） | （＋） | （＋） |
| 47 | 72 | M | Primary | T1 | low | （-） | （-） | （＋） |
| 48 | 71 | M | Primary | T2 | high | （-） | （-） | （＋） |
| 49 | 52 | M | Recurrent | T1 | low | （-） | （-） | （-） |
| 50 | 31 | M | Primary | T1 | low | （-） | （-） | （＋） |
| 51 | 72 | M | Primary | T1 | low | （-） | （-） | （-） |
| 52 | 69 | M | Recurrent | T1 | high | （-） | （-） | （＋） |
| 53 | 67 | M | Recurrent | T1 | high | （＋） | （＋） | （-） |
| 54 | 67 | M | Primary | T1 | high | （-） | （＋） | （-） |
| 55 | 66 | M | Primary | T1 | high | （-） | （-） | （-） |
| 56 | 63 | M | Primary | T1 | high | （-） | （＋） | （-） |
| 57 | 63 | M | Recurrent | T1 | high | （-） | （-） | （＋） |
| 58 | 80 | M | Primary | T1 | high | （-） | （-） | （＋） |
| 59 | 38 | M | Primary | T1 | high | （-） | （-） | （＋） |
| 60 | 62 | M | Primary | T1 | high | （-） | （-） | （＋） |
| 61 | 66 | M | Primary | T1 | high | （-） | （-） | （-） |
| 62 | 65 | M | Recurrent | T1 | high | （＋） | （＋） | （-） |
| 63 | 76 | M | Primary | T1 | high | （-） | （＋） | （＋） |
| 64 | 61 | M | Primary | T3 | high | （＋） | （＋） | （＋） |
| 65 | 46 | M | Recurrent | T2 | high | （＋） | （-） | （＋） |
| 66 | 75 | M | Primary | T3 | high | （-） | （-） | （＋） |
| 67 | 65 | M | Primary | T1 | high | （-） | （＋） | （＋） |
| 68 | 62 | M | Primary | T1 | high | （-） | （-） | （-） |
| 69 | 80 | M | Primary | T1 | high | （-） | （-） | （＋） |
| 70 | 56 | M | Primary | T1 | high | （-） | （-） | （-） |
| 71 | 70 | M | Primary | T1 | high | （-） | （-） | （＋） |
| 72 | 62 | M | Primary | T1 | high | （-） | （-） | （-） |
| 73 | 57 | M | Primary | T1 | high | （＋） | （＋） | （＋） |
| 74 | 55 | M | Primary | T1 | high | （-） | （-） | （-） |
| 75 | 79 | M | Primary | T3 | high | （-） | （-） | （-） |
| 76 | 70 | M | Primary | T1 | high | （-） | （＋） | （-） |
| 77 | 84 | M | Primary | T1 | high | （-） | （-） | （-） |
| 78 | 64 | M | Primary | T1 | high | （-） | （-） | （＋） |
| 79 | 53 | M | Primary | T1 | high | （-） | （-） | （-） |
| 80 | 74 | M | Recurrent | T1 | high | （＋） | （-） | （-） |
| 81 | 52 | M | Primary | T1 | high | （-） | （-） | （-） |
| 82 | 65 | M | Primary | T1 | low | （-） | （-） | （-） |
| 83 | 56 | M | Primary | T2 | high | （-） | （-） | （-） |
| 84 | 59 | M | Primary | T3 | high | （-） | （＋） | （＋） |
| 85 | 66 | M | Recurrent | T2 | low | （-） | （-） | （-） |
| 86 | 65 | M | Recurrent | T2 | high | （-） | （-） | （＋） |
| 87 | 59 | M | Primary | T1 | high | （-） | （-） | （-） |
| 88 | 74 | M | Primary | T1 | high | （-） | （-） | （-） |
| 89 | 64 | M | Primary | T1 | high | （-） | （＋） | （-） |
| 90 | 59 | M | Recurrent | T1 | high | （-） | （-） | （-） |
| 91 | 74 | M | Primary | T3 | high | （＋） | （-） | （＋） |
| 92 | 63 | M | Recurrent | T1 | high | （-） | （-） | （＋） |
| 93 | 58 | M | Primary | T1 | high | （-） | （＋） | （＋） |
| 94 | 63 | M | Primary | T2 | high | （-） | （-） | （-） |
| 95 | 55 | M | Primary | T2 | high | （-） | （-） | （＋） |
| 96 | 69 | M | Recurrent | T2 | high | （-） | （-） | （-） |
| 97 | 37 | M | Primary | T1 | low | （-） | （-） | （-） |
| 98 | 62 | M | Recurrent | T1 | high | （-） | （-） | （-） |
| 99 | 72 | F | Primary | T3 | high | （＋） | （-） | （-） |
| 100 | 74 | F | Primary | T1 | high | （-） | （-） | （-） |
| 101 | 75 | F | Recurrent | Ta | low | （＋） | （-） | （＋） |
| 102 | 91 | F | Primary | T1 | high | （-） | （-） | （＋） |
| 103 | 65 | F | Recurrent | T1 | low | （-） | （-） | （＋） |
| 104 | 52 | F | Primary | T2 | high | （-） | （-） | （＋） |
| 105 | 57 | F | Primary | T1 | high | （-） | （＋） | （-） |
| 106 | 54 | F | Primary | T1 | high | （-） | （＋） | （-） |
| 107 | 72 | F | Primary | Ta | low | （-） | （＋） | （-） |
| 108 | 54 | F | Primary | T1 | high | （-） | （-） | （-） |
| 109 | 56 | F | Primary | T1 | high | （-） | （＋） | （＋） |
| 110 | 71 | F | Primary | T1 | high | （-） | （-） | （-） |
| 111 | 37 | F | Primary | T1 | high | （-） | （-） | （-） |
| 112 | 76 | F | Primary | T2 | high | （-） | （-） | （-） |
| 113 | 55 | F | Primary | Ta | low | （-） | （-） | （-） |
| 114 | 58 | F | Primary | T1 | low | （＋） | （＋） | （＋） |
| 115 | 57 | F | Primary | T1 | high | （-） | （-） | （＋） |
| 116 | 74 | F | Primary | T1 | high | （＋） | （-） | （-） |
| 117 | 68 | F | Primary | Ta | high | （-） | （-） | （＋） |
| 118 | 55 | F | Primary | T1 | high | （-） | （-） | （＋） |
| 119 | 73 | F | Primary | T1 | high | （＋） | （-） | （-） |
| 120 | 93 | F | Primary | T1 | high | （-） | （-） | （＋） |
| 121 | 70 | F | Primary | T1 | high | （＋） | （＋） | （-） |
| 122 | 59 | F | Primary | T1 | high | （-） | （＋） | （-） |
| 123 | 51 | F | Primary | T1 | high | （-） | （-） | （＋） |
| 124 | 73 | F | Primary | T1 | high | （-） | （-） | （＋） |
| 125 | 62 | F | Primary | T3 | high | （-） | （-） | （＋） |
| 126 | 71 | F | Primary | T1 | high | （＋） | （＋） | （-） |
| 127 | 68 | F | Primary | T1 | high | （-） | （＋） | （-） |
| 128 | 80 | F | Primary | T1 | high | （-） | （+） | （＋） |
