# Supplementary material for: Urinary Exosomal Long Noncoding RNA TERC as a Noninvasive Diagnostic and Prognostic Biomarker for Bladder Urothelial Carcinoma
Source: J Immunol Res. 2022 Jan 25;2022:9038808. doi: 10.1155/2022/9038808 (PMC8811540; doi:10.1155/2022/9038808)
Supplement: Supplementary 2 — Supplementary File 2: the absolute levels of genes and NMP-22 (ELISA). [file 9038808.f2.docx]

**Ct Value in urine exosomes**

| Normal(n=94) | | Benign lesion(n=46) | | BLCA(n=128) | |
| --- | --- | --- | --- | --- | --- |
| TERC | 18S | TERC | 18S | TERC | 18S |
| 31.38 | 11.07 | 27.5 | 9.16 | 28.04 | 11.69 |
| 30.91 | 12.6 | 32.99 | 14.605 | 28.64 | 16.415 |
| 31.47 | 11.63 | 28.36 | 9.605 | 33.88 | 16.925 |
| 27.77 | 10.79 | 30.04 | 11.115 | 27.61 | 12.845 |
| 33.13 | 11.88 | 31.55 | 12.14 | 26.76 | 10.335 |
| 31.32 | 12.61 | 30.98 | 12.7 | 25.95 | 8.615 |
| 30.98 | 12.89 | 28.06 | 9.92 | 29.64 | 13.04 |
| 33.43 | 16.875 | 31.4 | 12.005 | 26.87 | 13.1 |
| 29.91 | 11.435 | 31.17 | 11.595 | 26.12 | 13.85 |
| 31.98 | 13.15 | 29.82 | 11.79 | 28.14 | 9.44 |
| 30.22 | 12.225 | 28.35 | 10.405 | 25.48 | 6.9 |
| 30.72 | 11.88 | 29.57 | 11.6 | 32.23 | 13.72 |
| 28.86 | 10.865 | 31.19 | 13.12 | 33.41 | 14.65 |
| 28.74 | 9.685 | 30.9 | 12.02 | 30 | 13.53 |
| 28.62 | 9.845 | 27.56 | 9.03 | 29.95 | 11.98 |
| 31.87 | 12.95 | 29.03 | 11.85 | 32.49 | 15.74 |
| 29.7 | 11.85 | 30.35 | 11.265 | 30.75 | 13.97 |
| 31.97 | 12.95 | 28.39 | 10.195 | 30.16 | 12.83 |
| 27.84 | 12.73 | 30.16 | 13.495 | 29.27 | 14.82 |
| 29.99 | 13.73 | 29.49 | 11.39 | 32.23 | 16.07 |
| 30.87 | 13.64 | 30.1 | 12.825 | 27.99 | 12.73 |
| 31.47 | 11.385 | 27.73 | 9.88 | 30.33 | 12.09 |
| 31.4 | 12.365 | 29.26 | 11.61 | 29.56 | 10.04 |
| 30.01 | 11.875 | 32.18 | 12.85 | 27.28 | 12.71 |
| 32.44 | 12.855 | 31.6 | 12.785 | 27.24 | 13.14 |
| 28.45 | 10.28 | 29.8 | 9.95 | 30.75 | 14.11 |
| 31.95 | 13.735 | 27.62 | 9.95 | 29.94 | 13.5 |
| 32.38 | 15.99 | 32.2 | 13.955 | 27.83 | 12.88 |
| 27.29 | 11.17 | 29.55 | 12.705 | 24.01 | 7.41 |
| 32.95 | 14.385 | 33.9 | 14.88 | 29.47 | 9.98 |
| 32.95 | 13.61 | 30.29 | 11.76 | 28.7 | 11.72 |
| 31.02 | 11.595 | 29.5 | 11.81 | 27.57 | 11.35 |
| 32.81 | 15.835 | 29.76 | 14.765 | 29.95 | 12.73 |
| 32.77 | 11.685 | 28.36 | 12.91 | 32.97 | 13.7 |
| 33.56 | 13.86 | 30.55 | 12.815 | 28.5 | 13.655 |
| 29.3 | 12.895 | 29.88 | 12.71 | 32.15 | 13.845 |
| 31.93 | 11.935 | 29.86 | 11.745 | 27.34 | 14.62 |
| 31.33 | 11.705 | 28.32 | 10.845 | 25.97 | 12.86 |
| 29.93 | 10.06 | 29.95 | 12.01 | 30.16 | 11.625 |
| 28.92 | 9.85 | 32.13 | 15.975 | 28.35 | 12.7 |
| 29.12 | 10.74 | 27.26 | 9.96 | 25.59 | 10.755 |
| 32.87 | 13.555 | 29.47 | 11.635 | 30.76 | 12.065 |
| 24.58 | 5.57 | 28.33 | 10.815 | 29.65 | 11.585 |
| 27.55 | 9.175 | 31.09 | 13.415 | 28.67 | 10.16 |
| 29.69 | 9.865 | 28.21 | 10.315 | 27.76 | 10.3 |
| 31.13 | 12 | 29.8 | 10.755 | 28.51 | 8.94 |
| 28.98 | 10.175 |  |  | 31.98 | 11.195 |
| 30.27 | 11.68 |  |  | 32.24 | 13.22 |
| 30.09 | 11.83 |  |  | 32.82 | 14.665 |
| 29.1 | 10.88 |  |  | 31.04 | 11.685 |
| 30.82 | 11.53 |  |  | 29.57 | 11.675 |
| 28.93 | 10.04 |  |  | 29.06 | 10.92 |
| 30.91 | 10.89 |  |  | 28.04 | 11.69 |
| 30.84 | 11.605 |  |  | 30.23 | 12.045 |
| 29.69 | 9.845 |  |  | 30.87 | 12.48 |
| 30.99 | 11.01 |  |  | 26.9 | 13.915 |
| 29.8 | 10.98 |  |  | 32.33 | 13.4 |
| 31.46 | 10.245 |  |  | 30.05 | 11.02 |
| 32.87 | 12.7 |  |  | 27.84 | 10.415 |
| 29.76 | 9.86 |  |  | 29 | 12.91 |
| 28.91 | 10.015 |  |  | 32.21 | 13.35 |
| 31.33 | 12.255 |  |  | 28.82 | 14.405 |
| 30.53 | 10.2 |  |  | 32.04 | 13.96 |
| 30.19 | 10.785 |  |  | 27.73 | 10.475 |
| 30.73 | 11.02 |  |  | 30.1 | 15.255 |
| 27.56 | 8.905 |  |  | 28.63 | 12.855 |
| 32.79 | 15.96 |  |  | 31.38 | 12.725 |
| 29.98 | 11.57 |  |  | 31.8 | 17.555 |
| 31.01 | 13.705 |  |  | 31.46 | 13.485 |
| 30.35 | 13.515 |  |  | 32.19 | 12.71 |
| 29.05 | 10.845 |  |  | 30.73 | 11.98 |
| 29.86 | 10.97 |  |  | 29.2 | 11.37 |
| 28.52 | 11.5 |  |  | 30.26 | 11.965 |
| 28.76 | 10.65 |  |  | 31.55 | 12.985 |
| 33.76 | 15.51 |  |  | 25.85 | 8.545 |
| 32.43 | 14.75 |  |  | 27.99 | 11.64 |
| 31.12 | 11.79 |  |  | 32.09 | 13.41 |
| 28.36 | 9.865 |  |  | 29.07 | 13.685 |
| 29.55 | 11.975 |  |  | 27.92 | 10 |
| 27.98 | 9.485 |  |  | 31.97 | 13.65 |
| 27.81 | 9.16 |  |  | 32.16 | 13.835 |
| 28.53 | 9.965 |  |  | 32.62 | 18.19 |
| 27.58 | 8.56 |  |  | 28.31 | 11.145 |
| 27.46 | 8.7 |  |  | 27.18 | 8.145 |
| 32.28 | 13.795 |  |  | 26.66 | 8.725 |
| 31.59 | 12.835 |  |  | 28.97 | 12.125 |
| 29.01 | 13.09 |  |  | 29.05 | 12.175 |
| 30.15 | 14.175 |  |  | 28.4 | 11.19 |
| 30.2 | 11.075 |  |  | 28.48 | 11.775 |
| 31.58 | 13.9 |  |  | 28.52 | 13.015 |
| 31.98 | 14.025 |  |  | 28.71 | 13.525 |
| 31.48 | 11.655 |  |  | 27.63 | 12.11 |
| 31.38 | 14.73 |  |  | 28.01 | 10.175 |
| 33.39 | 13.805 |  |  | 28.55 | 13.05 |
|  |  |  |  | 29.29 | 13.05 |
|  |  |  |  | 28.14 | 10.52 |
|  |  |  |  | 27.61 | 9.9 |
|  |  |  |  | 28.55 | 12.965 |
|  |  |  |  | 28.1 | 11.04 |
|  |  |  |  | 28.7 | 13.645 |
|  |  |  |  | 28.36 | 12.195 |
|  |  |  |  | 24.09 | 6.25 |
|  |  |  |  | 29.38 | 14.03 |
|  |  |  |  | 28.45 | 10.595 |
|  |  |  |  | 27.71 | 10.93 |
|  |  |  |  | 29.05 | 12.25 |
|  |  |  |  | 25.16 | 8.115 |
|  |  |  |  | 25.93 | 11.03 |
|  |  |  |  | 28.2 | 11.555 |
|  |  |  |  | 28.63 | 13.94 |
|  |  |  |  | 28.37 | 12.005 |
|  |  |  |  | 28.71 | 14.02 |
|  |  |  |  | 29.05 | 12.32 |
|  |  |  |  | 28.2 | 11.175 |
|  |  |  |  | 28.38 | 13.615 |
|  |  |  |  | 28.36 | 14.105 |
|  |  |  |  | 28.98 | 15.175 |
|  |  |  |  | 28.72 | 13.02 |
|  |  |  |  | 29.07 | 12.145 |
|  |  |  |  | 28.86 | 12.96 |
|  |  |  |  | 27.9 | 13.615 |
|  |  |  |  | 28.36 | 12.475 |
|  |  |  |  | 28.72 | 12.15 |
|  |  |  |  | 29.07 | 12.15 |
|  |  |  |  | 28.45 | 12.71 |
|  |  |  |  | 27.83 | 10.145 |
|  |  |  |  | 28.45 | 13.465 |
|  |  |  |  | 27.37 | 9.675 |

**Ct Value in tissues**

| Normal(n=23) | | BLCA(n=39) | |
| --- | --- | --- | --- |
| TERC | GAPDH | TERC | GAPDH |
| 23.995 | 19.14 | 25.91 | 21.34 |
| 24.9503 | 20.866 | 28.49 | 25.797 |
| 27.085 | 23.263 | 26.305 | 19.337 |
| 29.615 | 26.83 | 20.761 | 17.21 |
| 27.551 | 26.515 | 24.715 | 20.826 |
| 27.267 | 23.717 | 27.533 | 26.043 |
| 30.265 | 26.02 | 28.378 | 26.92 |
| 29.537 | 28.523 | 23.433 | 21.507 |
| 30.97 | 26.173 | 23.582 | 19.272 |
| 33.083 | 29.553 | 31.245 | 23.253 |
| 27.757 | 20.173 | 26 | 24.71 |
| 30.46 | 25.627 | 28.83 | 27.577 |
| 22.61 | 20.375 | 24.923 | 25.413 |
| 19.835 | 16.325 | 25.49 | 23.725 |
| 30.93 | 27.725 | 19.345 | 17.105 |
| 19.57 | 18.36 | 25.875 | 22.725 |
| 25.77 | 24.2 | 21.945 | 18.35 |
| 19.335 | 17.46 | 18.83 | 16.37 |
| 20.26 | 16.255 | 27.195 | 27.405 |
| 28.52 | 24.945 | 26.83 | 23.83 |
| 31.61 | 29.355 | 26.21 | 23.945 |
| 25.97 | 22.205 | 26.07 | 25.78 |
| 31.845 | 28.505 | 28.98 | 25.99 |
|  |  | 21.775 | 19.69 |
|  |  | 20.69 | 17.36 |
|  |  | 17.37 | 16.59 |
|  |  | 22.855 | 20.46 |
|  |  | 18.46 | 18.26 |
|  |  | 22.7 | 19.99 |
|  |  | 29.335 | 29.37 |
|  |  | 26.675 | 25.84 |
|  |  | 27.055 | 26.8 |
|  |  | 26.835 | 24.75 |
|  |  | 21.515 | 16.3 |
|  |  | 23.61 | 17.54 |
|  |  | 18.715 | 15.27 |
|  |  | 22.25 | 18.13 |
|  |  | 18.625 | 16.76 |
|  |  | 17.355 | 14.54 |

**NMP-22 (ELISA)**

| Normal(n=63) | BLCA(n=89) | U/ml |
| --- | --- | --- |
| 11.04807692 | 14.07692308 |  |
| 7.682692308 | 24.125 |  |
| 7.875 | 14.60576923 |  |
| 21.24038462 | 15.23076923 |  |
| 11.33653846 | 13.40384615 |  |
| 14.55769231 | 13.78846154 |  |
| 10.32692308 | 17.25 |  |
| 10.13461538 | 13.59615385 |  |
| 11.76923077 | 21.24038462 |  |
| 15.61538462 | 21.91346154 |  |
| 18.74038462 | 19.84615385 |  |
| 14.89423077 | 7.875 |  |
| 17.25 | 10.71153846 |  |
| 18.32638889 | 6.576923077 |  |
| 20.56730769 | 6.096153846 |  |
| 13.06730769 | 11.04807692 |  |
| 10.95192308 | 10.80769231 |  |
| 11.96153846 | 13.01923077 |  |
| 13.83653846 | 19.31730769 |  |
| 12.49038462 | 11.19230769 |  |
| 15.32692308 | 13.98076923 |  |
| 21 | 6.096153846 |  |
| 17.73076923 | 9.846153846 |  |
| 14.19444444 | 3.692307692 |  |
| 16.17361111 | 7.105769231 |  |
| 23.69230769 | 4.990384615 |  |
| 17.20192308 | 6.240384615 |  |
| 16.48076923 | 7.25 |  |
| 17.25 | 10.18269231 |  |
| 16.38461538 | 10.47115385 |  |
| 11.97222222 | 15.13461538 |  |
| 8.8125 | 7.875 |  |
| 4.125 | 6.480769231 |  |
| 5.131944444 | 6.625 |  |
| 14.71527778 | 7.730769231 |  |
| 30.0625 | 7.346153846 |  |
| 12.59722222 | 12.39423077 |  |
| 20.09722222 | 7.682692308 |  |
| 23.98611111 | 6.625 |  |
| 18.22222222 | 3.788461538 |  |
| 11.48611111 | 12.14583333 |  |
| 14.26388889 | 7.111111111 |  |
| 1.243055556 | 16.79861111 |  |
| 20.93055556 | 23.88194444 |  |
| 14.64583333 | 21.24305556 |  |
| 17.11111111 | 13.46527778 |  |
| 14.125 | 4.958333333 |  |
| 7.111111111 | 2.805555556 |  |
| 7.215277778 | 3.881944444 |  |
| 12.52777778 | 1.208333333 |  |
| 4.298611111 | 7.944444444 |  |
| 8.118055556 | 20.47916667 |  |
| 7.840277778 | 18.46527778 |  |
| 12.18055556 | 15.16666667 |  |
| 19.95833333 | 18.53472222 |  |
| 17.59722222 | 9.645833333 |  |
| 12.25 | 16.13888889 |  |
| 12.59722222 | 8.222222222 |  |
| 18.39583333 | 7.666666667 |  |
| 11.79861111 | 13.98611111 |  |
| 5.861111111 | -1.222222222 |  |
| 22.28472222 | 9.159722222 |  |
| 11.83333333 | 7.840277778 |  |
|  | 18.25694444 |  |
|  | 11.52083333 |  |
|  | -0.458333333 |  |
|  | 17.38888889 |  |
|  | 11.90277778 |  |
|  | 7.041666667 |  |
|  | 9.402777778 |  |
|  | -0.423611111 |  |
|  | 0.097222222 |  |
|  | 3.361111111 |  |
|  | -1.361111111 |  |
|  | 3.256944444 |  |
|  | 11.27777778 |  |
|  | 11.90277778 |  |
|  | 13.01388889 |  |
|  | 12.77083333 |  |
|  | 9.4375 |  |
|  | 3.847222222 |  |
|  | 5.895833333 |  |
|  | 5.236111111 |  |
|  | 2.25 |  |
|  | -2.368055556 |  |
|  | 1.972222222 |  |
|  | 13.01388889 |  |
|  | 6.381944444 |  |
|  | 0.618055556 |  |
